# Effect of the Task Approach, Airway Clearance, Kinesthetic Exercise (TASKS) Paediatric Rehabilitation Protocol on Miller-Fisher Syndrome Variant of Guillain-Barré Syndrome: A Case Report

**DOI:** 10.7759/cureus.57391

**Published:** 2024-04-01

**Authors:** Pratiksha A Warghat, H V Sharath, Sakshi Desai

**Affiliations:** 1 Department of Paediatric Physiotherapy, Ravi Nair Physiotherapy College, Datta Meghe Institute of Higher Education & Research (Deemed to be University), Wardha, IND

**Keywords:** miller-fisher syndrome, gbs variant, cardiovascular and pulmonary physiotherapy, paediatric rehabilitation, guillain-barré syndrome

## Abstract

Miller-Fisher syndrome (MFS) is a rare variant of Guillain-Barré syndrome (GBS) with varying incidence rates geographically. MFS is primarily diagnosed based on clinical features, such as ataxia and areflexia, although other neurological symptoms may also present. A case of MFS has been presented, characterized by complaints of ataxia, areflexia, bilateral foot and hand pain and difficulty in swallowing. In this regard, a paediatric rehabilitation approach has been adopted, utilizing outcome measures, such as the Erasmus Guillain-Barre Syndrome Respiratory Insufficiency Score-Kids, WeeFIM and paediatric balance scale, in addition to clinical evaluation. It is worth noting that the presented case demonstrates the importance of accurately diagnosing and treating this rare neurological condition MFS. Through the implementation of appropriate rehabilitation strategies, it is possible to enhance patients' quality of life.

## Introduction

Miller-Fisher syndrome (MFS) manifests as a sudden and self-limiting disorder distinguished by three primary clinical indications: paralysis of the eye muscles (ophthalmoplegia), loss of muscle coordination (ataxia) and absence of reflexes (areflexia). This constellation of symptoms was initially identified by James Collier in 1932 and later recognized as a variant of Guillain-Barré syndrome (GBS) by Charles Miller Fisher in 1956. Fisher noted the distinctiveness of these clinical features and their association with immune-mediated neuropathies under the GBS umbrella. Most cases of MFS are preceded by an antecedent infectious illness, commonly linked to pathogens, like *Campylobacter jejuni* and *Haemophilus influenza*, although others, such as *Mycoplasma pneumonia* and cytomegalovirus, are also implicated, with upper respiratory and gastrointestinal infections being frequently reported [1-4]

The Fisher variant of GBS (Fisher syndrome), known as MFS, has significantly contributed to understanding the pathogenesis of immune-mediated neuropathies, postulated to arise from molecular mimicry following prior infections. The presence of anti-GQ1b antibodies, identified by Chiba et al. in 1992, is closely associated with MFS, serving as a crucial diagnostic marker. These antibodies, detected in over 90% of affected individuals, play a diagnostic role in MFS and are also implicated in other forms of GBS affecting ocular muscles. Research indicates molecular similarities between peripheral nerve and microbial/viral antigens, which likely trigger autoimmune responses involving humoral and cell-mediated lymphocytes [5,6].

Treatment typically involves plasmapheresis and intravenous immunoglobulin (IVIG) for GBS, although specific considerations, such as ICU admission and mechanical ventilation, may arise before initiating physiotherapy. MFS typically presents with acute neurological symptoms approximately eight to 10 days after a preceding illness, progressing until a clinical nadir is reached around six days after symptom onset. Subsequently, a gradual recovery ensues with a resolution of symptoms, although rare complications, like respiratory failure or cardiac arrhythmias, common in GBS, can occur. Ataxia and ophthalmoplegia typically resolve within one to three months, with complete recovery expected within six months, except for persistent areflexia not associated with functional disability [7,8]. The main goals of the Task Approach, Airway Clearance, Kinesthetic Exercise (TASKS) Protocol in MFS rehabilitation are to improve strength, mobility, coordination and balance, enhance sensory-motor integration, provide functional training, teach adaptive strategies, monitor progress and deliver patient-centred care for improved daily functioning and quality of life.

## Case presentation

We present the case of a 10-year-old male child who came to the Acharya Vinoba Bhave Rural Hospital (AVBRH) Paediatric Department. As mentioned by the father, the child had been healthy until seven days ago. Suddenly, he started experiencing a low-grade fever spike without chills and rigour, which was relieved by medication. However, a few days later, he developed a productive cough with white sputum in small quantities, which was associated with a cold. After a few days, he started experiencing pain in his foot and hand region, which was initially mild but gradually intensified. As the days passed, the child's condition worsened, and he found it difficult to swallow solids than liquids food. This was associated with pooling and drooling of saliva from the mouth. Consequently, the child was taken to the government hospital in Yavatmal, where he was diagnosed with GBS. Due to the severity of the child's condition, he was immediately put on oxygen support as he started experiencing difficulty breathing. Various medical investigations (X-ray, arterial blood gas (ABG) and nerve conduction velocity (NCV)) were conducted, and he was diagnosed with GBS. For further medical management, he was transferred to AVBRH, where he was admitted to the paediatric intensive care unit (PICU). The child's condition was critical, and he was intubated with an endotracheal tube (ET) as he was tachypnoeic, which was not subsiding with an oxygen mask. The mode of ventilation was pressure control, FIO_2_ 100% and positive end-expiratory pressure (PEEP) 5 cmH_2_O. Immediately, immunoglobulin therapy was initiated, which is the treatment of choice for GBS. In addition, physiotherapy sessions were also started on the same day to help the child to recover faster. The child's recovery was slow but steady, and he remained in the PICU for several days

Clinical presentation

Consent and informed assent were taken from both the child's parents and directly from the child before the assessment. On observation, the child was found to be conscious, cooperative and oriented to time, place and person with stable haemodynamic and ectomorphic build. The child was on mechanical ventilation via an ET tube, the mode of ventilation was pressure control, FIO_2_ was 100% and PEEP was 5 cmH_2_O. On respiratory examination, crackles were present in the bilateral lung field. On sensory examination, all the sensations (touch, pain, temperature and vibration) were intact in the bilateral upper and lower limbs.

Motor examination

Motor examination tone was normal, and power was reduced bilaterally for the upper and lower limbs. Muscle strength of the bilateral upper limb and lower limb was graded according to the Medical Research Council (MRC) grading system (Table 1).

**Table 1:** Manual muscle testing 0: no contraction; 1: flickering contraction; 2: partial ROM with gravity-eliminated position; 3: full ROM against gravity; 4: full ROM against gravity with moderate resistance; 5: full ROM against gravity with maximum resistance. ROM: range of motion

Muscles	Right	Left
Shoulder flexors	4/5	4/5
Shoulder extensors	4/5	4/5
Shoulder abductors	4/5	4/5
Shoulder adductors	4/5	4/5
Elbow flexors	4/5	4/5
Elbow extensors	4/5	4/5
Wrist flexors	4/5	4/5
Wrist extensors	4/5	4/5
Hip flexors	4/5	4/5
Hip extensors	4/5	4/5
Hip abductors	4/5	4/5
Hip adductors	4/5	4/5
Knee flexors	4/5	4/5
Knee extensors	4/5	4/5
Ankle dorsiflexors	4/5	4/5
Ankle plantar flexors	4/5	4/5

Reflexes are involuntary responses to stimuli, typically assessed by tapping specific areas of the body with a reflex hammer. In the described case, bilateral upper limbs are observed to have a normal response. This means that when the appropriate tendon is tapped with the reflex hammer, such as the biceps tendon in the arm or the triceps tendon at the back of the elbow, the muscles respond promptly and appropriately, demonstrating a typical reflex arc. Bilateral lower limbs are noted to be slightly reduced. This suggests that when the corresponding tendons in the legs are tapped, such as the patellar tendon just below the kneecap or the Achilles tendon at the back of the ankle, the muscle response is somewhat diminished compared to what is considered normal. This reduction in reflex response could indicate a slight impairment in the nerve pathways responsible for transmitting the reflex signal, as mentioned in Table 2.

**Table 2:** Deep tendon reflex of the bilateral upper limb and lower limb. 0: absent (areflexia); 1+: diminished (hyporeflexia);  2+: normal response; 3+ : exaggerated (brisk), 4+ : clonus

Deep tendon reflex	Left	Right
Bicep jerk	2+	2+
Triceps jerk	2+	2+
Knee jerk	1+	1+
Ankle jerk	1+	1+
Plantar reflex	Flex	flex

In the context of paediatric rehabilitation for a patient with MFS, a variant of GBS, a thorough examination of cranial nerves is essential. Cranial nerve glossopharyngeal and vestibulocochlear nerves were affected, and the rest of other cranial nerves are intact.

Intervention

**Figure 1:**
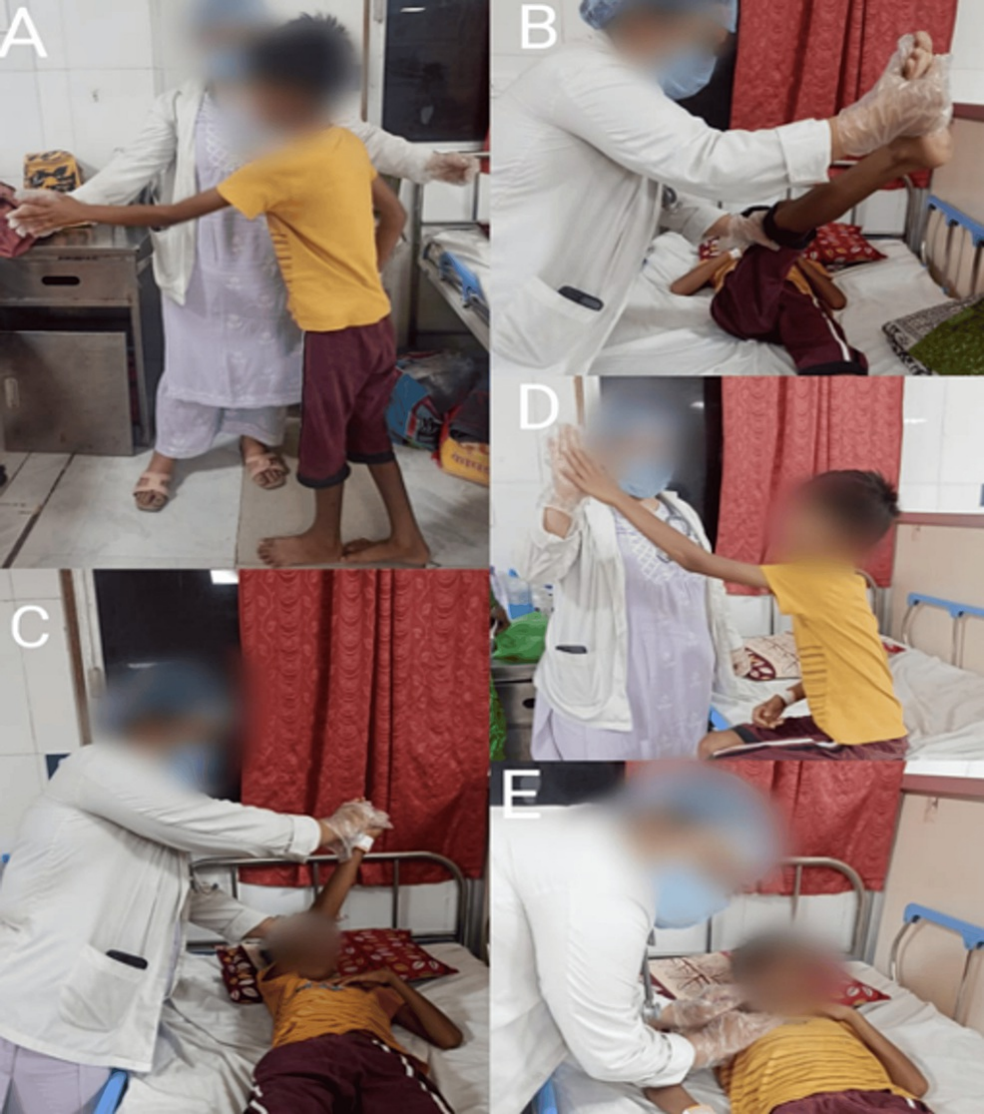
Patient performing exercises A: balance training using a task approach; B, C: proprioceptive neuromuscular facilitation techniques for the upper limb and lower limb; D: sitting balance with multidirectional reach-out task; E: chest clearance techniques

We made a tailored TASKS treatment protocol, which includes according to stages and symptoms, which includes the goals and intervention description [9], mentioned in Figure 1 and Table 3.

**Table 3:** TASKS physiotherapeutic intervention GBS: Guillain-Barre syndrome

Stages	Symptoms	Goals	Physiotherapy interventions	Procedure
Stage I (one month)	Secretion	Prevention of respiratory complication	AIRWAY CLEARANCE TECHNIQUES: Postural drainage with vibration and percussion techniques helps in the retention of secretion. This vibration percussion technique is provided to clear out the secretion.	Percussion procedure: It is a rhythmic tapping and clapping over the chest wall with one hand forming a cupped shape or three-fingered 'tenting' technique with the middle finger raised to overlap the first and third of the hand or infant face mask over the lung segment that is involved, which assist the mobilization of the bronchopulmonary secretion (at a rate of approximately 40/minute).
				Vibration position: Supine and side-lying vibrations are the oscillatory movements and intermittent chest wall compressions during expiration. We can effectively facilitate the movement of ribs and soft tissues in the direction of exhalation. The movement of vibration techniques is from peripheral to central over the chest wall. To perform vibrations, the therapist makes an oscillatory movement with their hands over the chest wall. The amount of pressure applied to the thorax is adjusted based on the patient's size, condition and the therapist's personal preference. This technique is started just before the patient exhales and can be continued until just before they inhale again These manual techniques can be used to help clear the airways of both large and small secretions. The goal is to move the secretions to a central location in the airway where they can either be coughed out or suctioned out.
				Proprioceptive neuromuscular facilitation techniques: Intercostal stretch is provided by applying pressure in between the intercoastal space of ribs, and the stretch is given in a downward (inward) direction in the intercoastal space. This procedure can be performed unilaterally or bilaterally.
				End-expiratory stretch: The patient positions in a supine lying position with the head end elevated. Hand placement is over the anterolateral part of the lower ribs, and then during end expiration, apply pressure over the lower ribs in inwards and downwards direction.
	Weakness of the muscles	For the activation of muscles	Task-oriented multimodal stimulation and proprioceptive neuromuscular facilitation (PNF) techniques are rehabilitation approaches that combine functionally based diagonal patterns of movement with neuromuscular facilitation to improve motor response, neuromuscular control and overall function. This approach is often used in physical therapy to help patients recover from injuries, improve mobility and range of motion and build strength and endurance. PNF techniques can involve a variety of exercises and movements, such as stretching, resistance training and dynamic movements.	Visual stimulation: including the task-appropriate approach. Various colourful toys, appropriate age-related vibrant colours animated picture books or visually stimulating games to incorporate child focus and participation during treatment. Use light and or rhyme videos, and play with different colourful torch lights. Movement tracking exercise in multi-directional gives task for a child to track the light with a torch light or colourful toys; this enhances eye movements, coordination and visual perception. Auditory stimulation: First, start with calling out the child's name, ask parents to talk to her and try to tell her stories; this stimulates auditory stimulation. Use soothing music, and play rhymes.
				Upper limb: D1 flexion for the shoulder in flexion adduction and external rotation, scapula in elevation abduction and upward rotation, elbow flexion or extension, forearm supination, wrist flexion and radial deviation and finger and thumb flexion adduction. D1 extension for shoulder extension-abduction-internal rotation scapula depression, adduction, downward rotation elbow flexion or extension pronation forearm wrist extension, ulnar deviation fingers and thumb extension, abduction. D2 flexion shoulder flexion-abduction-external rotation; scapula Elevation, abduction, upward rotation; elbow flexion or extension, forearm supination wrist extension, radial deviation; finger and thumb extension, abduction. D2 extension shoulder extension-adduction internal rotation; scapula depression, adduction downward rotation; elbow flexion or extension; forearm pronation wrist flexion, ulnar deviation; finger and thumb flexion, adduction.
				Lower limb: D1 flexion, hip flexion adduction, external rotation; knee flexion or extension; ankle dorsiflexion, inversion; toes extension D1 extension; hip extension abduction internal rotation; knee flexion or extension; ankle plantarflexion, eversion toe flexion. D2 flexion hip flexion abduction internal rotation; knee flexion or extension; ankle dorsiflexion, eversion; toe extension. D2 extension, hip extension adduction external rotation; knee flexion or extension; ankle plantar flexion inversion; toe flexion.
Stage II (two to three months)	Secretion and weak respiratory muscle	To clearance of airway	Airway clearance techniques: huffing and coughing techniques	Coughing: Coughing is a process that involves exhaling forcefully while keeping the glottis closed. This increases the pressure inside the chest. When the glottis opens, there is a sudden pressure difference between the smallest airways and the upper trachea, causing air to flow quickly. Due to the narrowing of the airways, the air's force increases, which helps to dislodge mucus and foreign particles that may be present in the airway. As a result, the mucus and foreign particles are pushed into the pharynx, which allows them to be expelled from the body. Sit upright or slightly reclined. Inhale deeply through your nose, hold your breath, and then exhale forcefully through your mouth using a cough. Repeat two to three times if necessary to clear your airways.
				Huffing: Huffing is a forced expiratory technique used to mobilize mucus from the bronchial tree. This manoeuvre involves maintaining an open glottis, generating lower intrathoracic pressure as compared to coughing. To perform the technique, one should sit up straight with the chin slightly tilted up and the mouth open. A slow, deep breath should be taken, filling the lungs about three-quarters full. After holding the breath for two to three seconds, one should exhale forcefully, but slowly and continuously. This compression and narrowing of the trachea and bronchi move secretions up from the bronchial tree and mobilize mucus from the smaller to the larger airways. The huffing technique is an effective method that promotes the clearance of mucus.
			Kinetic breathing	Kinetic breathing exercises: Controlled deep breathing exercises can help to increase the length and diameter of the airway and provide help to loosen bronchial secretions. Breathing exercise with movements: Incorporating gentle movements, such as arm or leg exercises, with coordinated breathing to enhance overall respiratory function. Position: sitting. Firstly, start with gentle shallow breathing and then ask the patient to take a deep breath through the nose and blow through the mouth (try to blow a candle or paper). Then, simultaneously add the upper limb movement as the child tries to breathe in ask to move the arms in an upward direction towards flexion; as the patient breaths out, take the arms in a downward direction.
	Balance and coordination	To improve the balance and coordination	The TASK approach including the neuro-developmental treatment (NDT) approach improves activities of daily living and improves strength in children with GBS. The basic pattern of posture and movement, the righting reaction and equilibrium responses are elucidated by providing the appropriate stimuli while abnormal patterns are inhibited. This approach also includes auditory and visual stimulation. Task approach: focusing on balance and coordination strategies.	Upper-extremity task: This improves the strength and range of motion of the upper extremities. Activities like picking up objects and holding and releasing objects of different sizes and textures. Drawing and colouring: Provide children with age-appropriate colouring books, crayons and markers. This activity promotes fine motor control, hand-eye coordination and creativity. Puzzle play: Engage children in puzzles with varying difficulty levels to enhance problem-solving skills and fine motor coordination. Craft activity: using paper try to make a paper boat or plane or craft. Pebble board activity that improves the grip of the hands.
				Lower-extremity task: Weight shifting while sitting or standing, holding the ball with both legs in sitting on a chair, initiating the ball kicking with softball and task-oriented sit-to-stand task. Various equipment and virtuality devices can be included. Play-based therapy: Utilizes play as a means of therapy, incorporating toys and games into rehabilitation activities.
				Play-based activities

Outcome measures 

Several outcome measures can be considered to assess the effectiveness of interventions to check the overall progress of the child. Here are some potential outcome measures that are relevant (pre and post intervention) (Table 4).

**Table 4:** Outcome measures The data has been represented as N. ERGIS-Kids: Erasmus Guillain-Barre Syndrome Respiratory Insufficiency Score-Kids EGRIS: Erasmus GBS Respiratory Insufficiency score 0 to 4: low risk of respiratory insufficiency, 5 to 8: moderate risk of respiratory insufficiency, 9 to 14: high risk of respiratory insufficiency. WeeFIM score 8-36: severe functional dependence; 37-54: moderate functional dependence; 55-72: mild to moderate functional dependence; 73-90: mild functional dependence; 91-108: near independence, 109-126: complete independence. Peadiatric Balance Scale score 0-20: severe balance impairment; 21-40: moderate balance impairment; 41-56: mild to no balance impairment

No.	Outcome measure	Pre-rehabilitation (/n)	Post-rehabilitation (/n)
1.	ERGIS-Kids	8/14	3/14
2.	Weefim	40/126	126/126
3.	Paediatric Balance Scale	03/56	44/56

## Discussion

This case report of paediatric rehabilitation on a child with MFS variant of GBS (MFS-GBS) presents a unique opportunity to discuss the challenges and successes in managing this rare neurological condition in children. Firstly, the presentation of MFS-GBS in paediatric patients often involves a distinct clinical profile characterized by ophthalmoplegia, ataxia and areflexia. These symptoms pose significant functional limitations and require a tailored rehabilitation approach to address mobility, balance and activities of daily living. This case underscores the importance of early recognition and multidisciplinary management to optimize outcomes in paediatric patients with MFS-GBS.

MFS is a rare subtype of GBS, a neurological disorder that affects the peripheral nervous system. It is characterized by a triad of symptoms, including ophthalmoplegia (paralysis of the eye muscles), ataxia (lack of coordination) and areflexia (diminished or absent reflexes). MFS is an autoimmune disorder, where the immune system mistakenly attacks the gangliosides (a type of glycolipid) in the peripheral nerves. This is thought to occur due to molecular mimicry, where antibodies generated against an infectious agent closely resemble gangliosides, leading to immune cross-reactivity. The diagnosis of MFS is primarily based on the clinical presentation and confirmed by the presence of anti-GQ1b antibodies, which are highly specific for the condition. These antibodies are found in up to 90% of MFS patients.

The rehabilitation process for MFS-GBS in children necessitates a comprehensive assessment of motor, sensory and cognitive functions to tailor interventions effectively [10,11]. This includes utilizing outcome measures, such as WeeFIM, muscle strength testing and gait analysis, to track progress and guide treatment planning. Given the potential for rapid deterioration in respiratory function, close monitoring and timely intervention are crucial to prevent respiratory compromise and ensure optimal outcomes in paediatric patients with MFS-GBS [12].

The case report highlights the integral role of a multidisciplinary team comprising physical therapists, occupational therapists, speech-language pathologists and other healthcare professionals in paediatric rehabilitation. Collaborative goal-setting and coordinated care delivery are essential to address the diverse needs of children with MFS-GBS, optimize functional outcomes and enhance the quality of life [13]. Moreover, involving the child and their caregivers in the rehabilitation process fosters empowerment, promotes adherence to treatment plans and facilitates the transition to home and community settings [14].

The management of MFS involves supportive care, immunomodulatory therapy and paediatric rehabilitation. IVIG is the preferred treatment with the physiotherapy approach from day one of the hospitalization [15-17]. The goal of treatment is to reduce the severity and duration of symptoms, prevent complications (such as respiratory failure or autonomic dysfunction) and facilitate recovery. Most patients with MFS experience spontaneous recovery within weeks to months, although some may require prolonged rehabilitation. Prompt recognition and management of the disorder are critical to improve outcomes and prevent complications. Paediatric rehabilitation plays a vital role in optimizing the child's functional outcomes and promoting recovery [18]. Physical therapy is a key component of paediatric rehabilitation, aimed at enhancing balance, coordination and strength, ultimately facilitating independent mobility.

The important aspect of paediatric rehabilitation focuses on fine motor skills and activities of daily living, while speech therapy targets any communication or swallowing difficulties that may arise. Close monitoring of respiratory function is of paramount importance, as respiratory compromise can occur in severe cases. IVIG therapy is an effective treatment option for paediatric patients with MFS. However, further research is necessary to determine the optimal dose and duration of treatment for this patient population [19,20]. The case report underscores the need for further research and clinical guidelines specific to paediatric MFS-GBS to optimize rehabilitation strategies and improve outcomes in this population. Longitudinal studies assessing the efficacy of different rehabilitation interventions, the impact on quality of life and predictors of recovery are warranted to inform evidence-based practice and enhance the standard of care for children with MFS-GBS. By sharing experiences and insights from clinical cases like this, healthcare professionals can contribute to advancing knowledge, improving outcomes and ultimately enhancing the quality of care for paediatric patients with MFS-GBS.

## Conclusions

MFS is a rare neurological disorder that primarily affects children. It is characterized by a triad of symptoms, including ophthalmoplegia (paralysis of the eye muscles), ataxia (loss of coordination) and areflexia (absence of reflexes). MFS poses significant challenges in the paediatric population that require a collaborative and multidisciplinary approach to ensure early recognition, prompt intervention and comprehensive rehabilitation. This approach involves a team of experts from paediatric neurology, rehabilitation and supportive care, who work together to maximize functional outcomes and improve the quality of life in children affected by this syndrome.
